# Linking structural and effective brain connectivity: structurally informed Parametric Empirical Bayes (si-PEB)

**DOI:** 10.1007/s00429-018-1760-8

**Published:** 2018-10-09

**Authors:** Arseny A. Sokolov, Peter Zeidman, Michael Erb, Philippe Ryvlin, Marina A. Pavlova, Karl J. Friston

**Affiliations:** 1 Wellcome Centre for Human Neuroimaging, Institute of Neurology, University College London (UCL), London, WC1N 3BG UK; 2 Service de Neurologie, Département des Neurosciences Cliniques, Centre Hospitalier Universitaire Vaudois (CHUV), 1011 Lausanne, Switzerland; 3 Department of Biomedical Magnetic Resonance, Department of Radiology, University of Tübingen Medical School, 72076 Tübingen, Germany; 4 Department of Psychiatry and Psychotherapy, University of Tübingen Medical School, 72076 Tübingen, Germany

**Keywords:** Effective connectivity, Dynamic causal modelling (DCM), Structural connectivity, Functional MRI

## Abstract

Despite the potential for better understanding functional neuroanatomy, the complex relationship between neuroimaging measures of brain structure and function has confounded integrative, multimodal analyses of brain connectivity. This is particularly true for task-related effective connectivity, which describes the causal influences between neuronal populations. Here, we assess whether measures of structural connectivity may usefully inform estimates of effective connectivity in larger scale brain networks. To this end, we introduce an integrative approach, capitalising on two recent statistical advances: Parametric Empirical Bayes, which provides group-level estimates of effective connectivity, and Bayesian model reduction, which enables rapid comparison of competing models. Crucially, we show that structural priors derived from high angular resolution diffusion imaging on a dynamic causal model of a 12-region network—based on functional MRI data from the same subjects—substantially improve model evidence (posterior probability 1.00). This provides definitive evidence that structural and effective connectivity depend upon each other in mediating distributed, large-scale interactions in the brain. Furthermore, this work offers novel perspectives for understanding normal brain architecture and its disintegration in clinical conditions.

**Electronic supplementary material:**

The online version of this article (10.1007/s00429-018-1760-8) contains supplementary material, which is available to authorized users.

## Introduction

Brain connectivity can be measured or inferred at multiple levels, but integrating these levels poses a significant challenge. *Effective connectivity* is the causal influence that neural populations exert over each other, and can be inferred from functional imaging data (e.g., functional MRI—fMRI, electroencephalography—EEG, or magnetoencephalography—MEG) via the inversion of forward or generative models (e.g., Dynamic Causal Modelling, DCM). Functional connectivity refers to the consequences of these causal interactions; for example, correlations between region- or source-specific time series. Between-region communication is mediated by direct axonal connections and, therefore, depends on the white-matter architecture of the brain. In neuroimaging, white matter or structural connectivity is typically characterised using diffusion MRI (dMRI; Jones et al. 1999; Mori and Zhang 2006; Jbabdi et al. 2015) and probabilistic tractography (Behrens et al. 2003). Given that brain function arises from the underlying network structure, a meaningful description of effective and functional connectivity should benefit from taking structure into account (Sporns et al. 2000; Park and Friston 2013; Sporns 2014). Indeed, studies comparing fMRI and dMRI measures for circumscribed connections or networks suggest that functional and effective connectivity generally reflect underlying anatomy (Upadhyay et al. 2008; Greicius et al. 2009; Saur et al. 2010; Ethofer et al. 2011; Sokolov et al. 2012, 2014). However, despite the potential benefits of integrative analyses, methodological challenges have limited the uptake of multimodal approaches, in particular with respect to larger scale brain networks.

The mapping between brain structure and function is not straightforward. Two regions may lack direct structural (axonal) connections, but, nonetheless, communicate through polysynaptic white-matter pathways (Koch et al. 2002). Furthermore, specific task demands modulate the extent to which particular brain regions are engaged. In other words, some contexts may silence effective connectivity despite the presence of structural connectivity (e.g., silent synapses; Isaac et al. 1995). Differences in spatial and temporal resolution between MRI, EEG, and MEG represent another significant challenge. It is, therefore, unsurprising that there are inconsistent findings across studies seeking to bridge structural and functional brain connectivity. Straightforward associations have been reported between structural connectivity and fMRI- or MEG-based resting-state functional connectivity (rsFC; Garces et al. 2016), as well as between white-matter fibre pathway characteristics and functional connection strength (Hermundstad et al. 2013), while other work has indicated a rather complex relationship, with existence of rsFC in the absence of detectable direct structural connections (Honey et al. 2009).

To accommodate this complex relationship between white-matter structure and brain communication, probabilistic approaches appear useful. For example, one might predict stronger functional or effective connectivity between regions with stronger structural connections. Recent studies have assessed the utility of probabilistic tractography as probabilistic priors for fMRI rsFC in Bayesian frameworks (Xue et al. 2015; Kang et al. 2017), and to form a prior precision (inverse variance) matrix used for MEG rsFC (Pineda-Pardo et al. 2014). However, without a model of neuronal coupling, these functional connectivity analyses cannot characterise the causal influences between brain regions; namely, their effective connectivity.

**Fig. 1:**
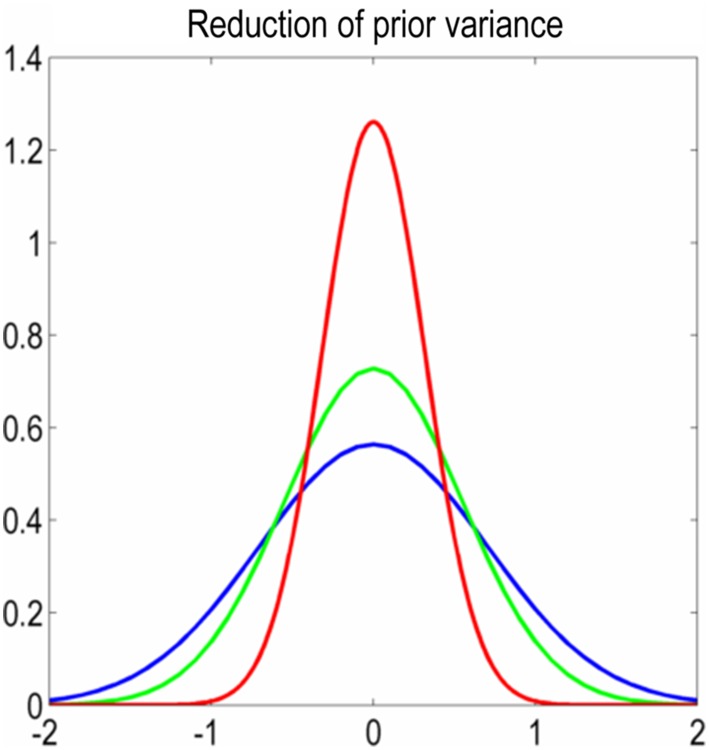
Illustration of shrinkage priors in DCM and their reduction. The horizontal axis is the value of the connectivity parameter (i.e., the strength of the connection) and the vertical axis is the prior probability for effective connectivity. The blue line (${\Sigma _{y~\hbox{max} }}$ = 0.5) is the maximum prior variance used in this study for extrinsic (between-region) DCM connections. Reducing the prior variance, illustrated by the green line (${\Sigma _y}$ = 0.3) and the red line (${\Sigma _y}$ = 0.1), limits the extent to which a posterior connection parameter can deviate from its prior expectation of zero

The added value of structural connectivity constraints on effective connectivity was previously demonstrated in the context of a four-node neural network (Stephan et al. 2009). The authors used circuitry models (DCMs), where the strength of every between-region connection is controlled by a parameter. These parameters have a prior (multivariate normal) distribution, with an expectation of zero and a certain positive variance. The larger the prior variance, the further the extrinsic effective connectivity is allowed to deviate from zero (Fig. 1). The authors specified a set of models which differed in the mapping from structural connectivity to prior variance, and they found that the model with the strongest evidence had a positive monotonic mapping from structural connectivity to the prior variance of extrinsic effective connectivity. While conceptually promising, the computational cost of estimating every DCM of effective connectivity with different structure–function mappings may have limited the uptake of this approach, particularly with regard to networks with larger numbers of nodes.

In the present study, we use a computationally efficient approach to ask whether structural information—afforded by probabilistic tractography on high angular resolution diffusion imaging (HARDI)—can be used to improve analyses of group-level extrinsic effective connectivity [i.e., the parameters that constitute the DCM A-matrix, see Eq. (2)]. In our approach, a single DCM is estimated for each subject in the usual way. Subsequently, a group-level Parametric Empirical Bayes (PEB) model is estimated, which takes the form of a Bayesian General Linear Model (GLM) of the subjects’ connection strengths and includes (parametric) random effects on connectivity (Friston et al. 2016). Using Bayesian model reduction (BMR), this ‘full’ PEB model is then analytically compared against hundreds of alternative models, which differ in the form of the mapping from structural connectivity to the prior variance of effective connectivity (Friston et al. 2016).

Formally, this procedure adds an extra hierarchical level to the model of observed functional time series. At the first level, the data are explained in terms of haemodynamics playing out on a network parameterised in terms of effective connectivity. At the second level, the connectivity parameters are generated by a group mean plus some random fluctuations (i.e., effects). At the final level, group means are generated under structural constraints; namely, departures of effective connectivity from their prior mean of zero depend upon the probability of a structural connection (Fig. 1). This dependency is itself parameterised. The use of Bayesian model reduction (BMR) to identify the parameters of the mapping between structural and effective connectivity is the key advance here. BMR is sufficiently fast and efficient to explore all plausible mappings, thereby avoiding the local minima associated with simpler optimisation schemes.

In what follows, we illustrate assessment of structural constraints derived from HARDI data for DCMs of effective connectivity based on task-related fMRI in the same subjects. The code for performing these analyses is available in the freely available Statistical Parametric Mapping (SPM12; http://www.fil.ion.ucl.ac.uk/spm) software package implemented in Matlab (MathWorks, Inc., Sherbon, MA, USA).

## Methods

### Participants

fMRI and HARDI data of 12 right-handed male volunteers with a mean age of 26.0 years were included in this study. The participant group overlapped with that in previous studies (Sokolov et al. 2012, 2014). All participants had normal visual acuity and no history of neurological or psychiatric conditions or medication. Informed written consent was obtained and subjects were financially compensated for their participation. Study approval was obtained from the Ethics Committee of the University of Tübingen Medical School, Germany.

### Experimental setup

While undergoing fMRI, subjects viewed visual biological motion stimuli. These consisted of 11 bright dots on the head and main articulations of a person walking to the right without displacement against a dark background, and were masked by 33 additional bright dots moving in the same fashion (for more details, see Pavlova et al. 2007). Arrays of 44 bright dots that did not contain the point-light walker constituted control stimuli. Stimulus duration was 1000 ms and a fixation cross was shown between presentation of stimuli and during baseline. The animations were created with Cutting’s algorithm (Cutting 1978), and displayed using the software Presentation (Neurobehavioral Systems Inc., Albany, CA, USA) through projection on a screen outside the MRI scanner (3 T TimTrio, Siemens Medical Solutions, Erlangen, Germany; 12 channel head coil). Participants viewed the screen through a tilted mirror on the head coil. In a two-alternative forced choice paradigm, the participants had to decide whether a walker was present or not by pressing a button with their right index finger (button order counterbalanced between participants).

### MRI recording

Over two fMRI sessions (echo-planar imaging (EPI); 114 volumes, 56 axial slices, TR = 4000 ms, TE = 35 ms, in-plane resolution 2 × 2 mm^2^, slice thickness = 2 mm, 1 mm gap), 120 trials (60 trials per stimulus type) were presented to the participants. Each session lasted 456 s and contained an initial baseline epoch of 24 s and three event-related epochs of 120 s (20 trials each, the same number of walker-present and absent stimuli), followed by a baseline epoch of 24 s each. Stimulus onset intervals were jittered between 4000 and 8000 ms in steps of 500 ms and stimulus order was pseudo-randomised.

A three-dimensional T1-weighted magnetisation-prepared rapid gradient echo (MPRAGE) imaging data set (176 sagittal slices, TR = 2300 ms, TE = 2.92 ms, TI = 1100 ms, and voxel size = 1 × 1 × 1 mm^3^) was acquired as anatomical reference and a field map for later correction of magnetic field inhomogeneity. For HARDI, we employed a diffusion-sensitised spin EPI with isotropic resolution (54 axial slices, TR = 7800 ms, TE = 108 ms, slice thickness = 2.5 mm, matrix size = 88 × 88, and field of view = 216 mm) and 64 diffusion gradient directions (*b* value = 2600s/mm^2^). Per subject, two HARDI sessions were performed to improve consistency and sensitivity of diffusion parameter estimation. Per session, one volume without diffusion sensitisation (*b* value = 0 s/mm^2^) was recorded.

### fMRI analysis

After standard pre-processing using SPM12, including slice timing and realignment, unwarping, image co-registration, normalisation based on segmentation and spatial smoothing, a GLM was specified for fMRI analysis. The timing of all stimulus onsets (across both stimulus types) was concatenated over sessions and modelled with a single regressor. A parametric regressor modelled the presented stimulus type (positive for walker-present and negative for walker-absent displays). Regressors of no interest included six head motion parameters, white matter, and cerebrospinal fluid time series. Subsequently, event-related regressors were convolved with a hemodynamic response function. A high-pass filter with 1/256 Hz was applied and the error term was modelled as a mixture of a first-order autoregressive process with a coefficient of 0.2 and white noise.

Individual whole-brain images were created for the contrasts: task (activation vs. baseline), positive parametric regressor (walker-present vs. walker-absent trials), and the reverse (walker-absent vs. walker-present trials). Second-level random effects analysis was conducted on the individual contrast images, and regional activations were localised using the automated anatomical labelling in SPM (Tzourio-Mazoyer et al. 2002) and the NeuroSynth.org database (Yarkoni et al. 2011; http://neurosynth.org). Contrast images were thresholded at *p* < 0.05, family-wise error (FWE) corrected for multiple comparisons using random field theory.

### HARDI pre-processing and probabilistic tractography

Based on the fMRI analysis above, we identified eight regions differentially activated by the presence and absence of the point-light walker within the array of moving dots (seven regions exhibiting higher activation for walker-present vs. walker-absent stimuli and one region for the inverse contrast) and four regions activated, on average, across all conditions as compared to baseline. The coordinates of these regions are presented in Table 1. Spherical images with centre coordinates identical to the fMRI individual maxima for these 12 regions and an 8 mm radius (corresponding to the DCM node specifications, see below) were created for probabilistic tractography.

**Table 1:** MNI coordinates, *z* values and cluster sizes (in mm^3^) of the regions included in the DCM based on group-level SPM analysis (p < 0.05, FWE-corrected for multiple comparisons) for walker-present as compared to walker-absent stimuli, walker-absent vs. walker-present trials and the active condition (all stimulus presentation as compared to baseline)

Anatomical label	MNI coordinates			*z* value	Cluster size
	*x*	*y*	*z*		
Walker-present vs. walker-absent					
L middle temporal cortex					
R middle temporal cortex	46	− 68	0	5.95	624
R insula	36	24	2	5.86	432
L cerebellar lobule Crus I	− 36	− 54	− 28	5.78	296
R superior temporal sulcus (STS)	50	− 40	10	5.62	736
R fusiform gyrus (FFG)	42	− 56	− 14	5.48	384
R inferior frontal gyrus (IFG)	46	10	32	5.41	704
Walker-absent vs. walker-present					
L V6	− 6	− 72	− 34	5.61	896
Active (stimulation vs. baseline)					
L V3	− 32	− 84	12	5.98	552
R V1	18	− 94	0	5.95	472
L V1	− 12	− 96	0	5.91	632
R V3	30	− 84	22	5.80	272

All 12 regions were included in the subsequent DCM analysis

For HARDI pre-processing, we used the FMRIB’s Diffusion Toolbox (FDT) within the FMRIB Software Library (FSL5, Oxford Centre for Functional MRI of the Brain, UK, http://www.fmrib.ox.ac.uk/fsl). After brain extraction (Smith 2002), motion and eddy current correction, and co-registration with the anatomical reference image, the data were aligned to normalised Montreal Neurological Institute (MNI) space. For the latter two steps, the transformation parameters provided by the FMRIB Linear Image Registration Tool (FLIRT; Jenkinson et al. 2002) were employed to adjust gradient directions accordingly. Bayesian Estimation of Diffusion Parameters Obtained using Sampling Techniques with modelling of Crossing Fibres (BEDPOSTX; Behrens et al. 2007) on individual normalised data yielded diffusion parameters for each voxel.

Subsequently, each of the 12 brain regions was used as a seed in probabilistic tracking with crossing fibres (PROBTRACKX; Behrens et al. 2007; step length = 0.5 mm, number of steps = 2000, number of pathways = 5000, curvature threshold = 0.2, modified Euler integration) with the other nodes specified as classification targets (according to neuroanatomical evidence, no structural connections were assessed between the left cerebellar lobule VI and the 5 occipital regions: bilateral V1 and V3, and left V6; Buckner et al. 2011).

### Structural connection strength

For every pair of seed and target regions, averaging the values contained in every seed voxel (representing the number of streamlines from this voxel to the target region) provided a streamline average count from the seed to the target region. The anatomical plausibility of the resulting structural pathways was confirmed by inspection for each subject. As dMRI does not provide information about directionality—and probabilistic tractography is known to vary depending on which region is defined as seed and target—for any pair of regions, the streamline counts for each tractography direction were averaged and stored in a symmetric between-region structural connectivity matrix. The individual structural connectivity matrices were averaged at the group level to create a second-level matrix. Given the computational efficiency of PEB (see below) and the empirical optimisation of how structural measures constrain effective connectivity priors, no thresholding was applied to the structural connectivity matrix. The group-average connection strengths were scaled relative to the maximum group-average connection strength.

### Dynamic causal modelling (DCM)

For each subject, based on the fMRI data, a DCM was specified (with options: one-state, bilinear, deterministic, and mean-centred inputs). DCM is a framework for Bayesian modelling of brain dynamics, divided into two parts—a *neuronal* model and an *observation* model (Friston et al. 2003). The neuronal model captures the change in brain activity due to recurrent neuronal connections and experimental inputs:

1 $$\dot {z}=f(z,u,{\theta ^n}),$$

where the vector $z \in {{\mathbb{R}}^n}$ represents the mass neural activity of each of $n$ brain regions and $\dot {z}$ is the derivative of $z$ with respect to time. Time series $u$ are the experimental inputs and ${\theta ^n}$ are neuronal coupling parameters that determine the (effective) strength of connections within and between brain regions. Group coordinates for the 12 regions (Table 1) identified according to fMRI activation and included in probabilistic tractography (described above) were used to inform extraction of individual time series for DCM. They were extracted by computing the first eigenvariate of all activated voxels (*p* < 0.05, uncorrected) within a sphere of 8 mm radius centred on each individual maximum, found in every subject within 6 mm of the group activation coordinate.

The approximation of Eq. (1) used for a particular experiment depends on the available data, and the question the experimenter wishes to address. Here, we used the basic neuronal model in DCM for fMRI, which approximates $\dot {z}$ using a simple function (a Taylor approximation):

2 $$\dot {z}=\left( {A+\mathop \sum \limits_{j} {u_j}{B^{(j)}}} \right)z+Cu.$$

The parameter matrix $A \in {{\mathbb{R}}^{N \times N}}$ represents the effective connectivity within and between each of the $N$ regions. Our model included bidirectional connections between all 12 network nodes, except between the left cerebellar lobule VI and the occipital regions (bilateral V1 and V3, left V6), in correspondence with the structural connectivity matrix. Parameters ${B^{(j)}} \in {{\mathbb{R}}^{N \times N}}$ are the modulatory effects of experimental manipulation $j$ on each connection (only modelled for self-connections of the regions here, which control the intrinsic excitability of each region) and $C \in {{\mathbb{R}}^{N \times J}}$ is the direct driving influence of each of the $J$ experimental inputs on each region. In this network, the driving input (all stimuli vs. baseline) was modelled to enter bilateral V1.

The second part of the model describes the observations we would expect to measure in the fMRI scanner, given the response of the neuronal model:

3 $$y=g\left( {z,{\theta ^h}} \right)+\varepsilon ,$$

where ${\theta ^h}$ are the parameters of the observation model and observation noise $\varepsilon$ is modelled as zero mean additive noise. DCM for fMRI approximates this function using a hemodynamic model that incorporates the ‘Balloon’ model of neurovascular coupling, including changes in blood flow and subsequent blood oxygen-level-dependent (BOLD) response (Stephan et al. 2007).

In summary, putting together the neuronal and observational models within DCM, we have parameters $\theta =(A,B,C,{\theta ^h})$, all of which are estimated from the data. In what follows, for simplicity, we concentrate on the extrinsic connectivity parameters *A*, although the described statistical framework and procedures can be applied to all parameters.

### Prior variance

In a probabilistic (Bayesian) model $m$, every parameter has a prior probability $p(\theta |m)$, on which DCM estimation depends. We set the prior on parameter ${A_{q,r}}$ which represents the connection strength from region $r$ to region $q$ as follows:

4 $$p\left( {{A_{{\text{q,r}}}}{\text{|}}m} \right)=N\left( {{A_{{\text{q,r}}}};\,\,0,~{\Sigma _y}} \right),$$

where the prior variance was ${\Sigma _y}$ = 0.5. This prior variance is central to our methodology for multimodal integration, so we briefly reprise its interpretation. The prior variance limits the extent to which the posterior estimate of a connection’s strength can deviate from its prior expectation of 0 Hz. The priors for connections in DCM form a multivariate normal distribution and are usually set to the same value across all extrinsic (i.e., between region) connections (Friston et al. 2003). If we set the prior variance to a small positive number (e.g., 0.1 or 0.3, as shown by the red and green lines in Fig. 1), we express the belief that the effective connection is likely to be small or absent. If the prior variance is set to a larger value (such as 0.5, blue line in Fig. 1), then we are willing to entertain connection strengths further from zero, if this is sufficiently supported by the observed data. By setting this prior variance to be a function of structural connectivity, the model evidence can be improved (Stephan et al. 2009). The influence of priors on model evidence will be illustrated further below.

### Structurally informed Parametric Empirical Bayes (si-PEB)

In contrast to Stephan et al. (2009), we modulated the prior variance on extrinsic connections at the group level, using the PEB framework. This creates a hierarchical model, in which the average group connectivity acts as an empirical prior on the connectivity parameters of individual subjects (Friston et al. 2016). PEB can increase the robustness of the DCM to random effects, because the contribution of each individual connection to the group level is weighted by its estimated precision, meaning that subjects with inefficient estimators will contribute less to the group result than subjects, whose data enable precise estimates. Furthermore, using empirical (group level) priors on DCM parameters finesses local minima problems (Friston et al. 2016). The PEB model for extrinsic effective connectivity has the following form:

5 $$\begin{aligned} {y_i}=\,\, & \Gamma _{i}^{{\left( 1 \right)}}\left( {A_{i}^{{\left( 1 \right)}}} \right)+\varepsilon _{i}^{{\left( 1 \right)}}, \\ A_{i}^{{\left( 1 \right)}}=\,\, & {\Gamma ^{\left( 2 \right)}}\left( {{A^{\left( 2 \right)}}} \right)+{\varepsilon ^{\left( 2 \right)}}. \\ \end{aligned}$$

Here, the observed fMRI data ${y_i}$ for subject $i$ are generated by function $\Gamma _{i}^{{(1)}}$—the subject’s DCM with parameters $A_{i}^{{(1)}}$—and observation noise $\varepsilon _{i}^{{(1)}}$. The DCM parameters $A_{i}^{{(1)}}$ are themselves represented by a group or second-level model, written on the second line of the equation. The second-level function ${\Gamma ^{\left( 2 \right)}}$ (a GLM) has parameters (e.g., group-average connection strengths) ${A^{(2)}}$, plus between-subject random effects ${\varepsilon ^{\left( 2 \right)}}$ (see Supplementary Methods). In the si-PEB approach presented here, we modulate the prior variance ${\Sigma _y}~$ of the second-level parameters ${A^{(2)}}$, in analogy to Eq. (4):

6 $$p\left( {{A^{\left( 2 \right)}}{\text{|}}m} \right)=N\left( {{A^{\left( 2 \right)}};\,\,0,~{\Sigma _y}} \right).$$

Based on the previous findings (Koch et al. 2002; Honey et al. 2009; Stephan et al. 2009), we assumed a positive monotonic relationship between structure and function; i.e., greater group structural connection strength *φ* induces a higher second-level prior variance ${\Sigma _y}~$ for the corresponding extrinsic effective connectivity, using a logistic (sigmoid) function:

7 $${\Sigma _y}=\frac{{{\Sigma _{y~\hbox{max} }}}}{{1~+~\exp ~(\alpha ~ - ~\delta ~ \times ~\varphi )}},$$

where the hyperparameter ${\Sigma _{y~\hbox{max} }}$ is the maximal prior variance on second-level effective connectivity, *δ* determines the slope, and α is the point of inflection of the sigmoid. To find the optimal hyperparameter combination, we varied α in steps of 0.5 in the range between − 2 and 2, *δ* in steps of 2 in the range between 0 and 16, and ${\Sigma _{y~\hbox{max} }}$ between 0.1 and 0.5 in steps of 0.1, resulting in 405 different transformations, and thus second-level models of effective connectivity. Importantly, this range of hyperparameters included mappings which were flat (*δ* = 0)—these formed control (i.e., null) models, where the effective connectivity was not informed by the structural connectivity. These 405 structure–function mappings constituted competing structural constraints that entered as priors on mean effective connectivity. By treating each mapping as a model, we evaluated their evidence to search for the best mapping, as follows.

Our focus was on using structurally informed priors to identify group-level functional architectures. However, in principle, the same procedures can be applied to single subject data. Equation (7) was also used to assess the utility of structural information at the individual level. Although one could optimize the mapping on a per subject basis, we took the slightly more conservative approach of using the same mapping for all subjects based on the outcome of the si-PEB group analysis. This allowed us to create structurally informed DCMs for every subject and evaluate their evidence in relation with a full, structurally naive DCM. This analysis assessed the consistency of structural constraints, in terms of the reproducibility of model comparison over subjects.

### DCM and PEB estimation

We estimated a DCM for each subject, which identified the parameters $\theta =(A,B,C,{\theta ^h})$ from Eqs. (1) and (3) that maximized the quality of the model, as quantified by the model evidence. The model evidence is defined as the probability of observing the measured data $y$ given the model $m$, $p(y|m)$. DCM performs model estimation using variational Bayes under the Laplace approximation (variational Laplace). For all DCM parameters *θ * (we focus on the extrinsic connectivity parameters *A* in this study), this procedure replaces the complicated distribution of posterior parameters $p(A|y,m)$ with the simpler distribution $q(A|y,m)$ and approximates the model evidence by the negative variational free energy $F$, which is a lower bound on the log model evidence $\log p\left( {y{\text{|}}m} \right)$. This is given by

8 $$\begin{aligned} F\left( m \right)= & \mathop \iint \nolimits^{} q\left( A \right){\text{~}}\ln \frac{{p\left( {y,A{\text{|}}m} \right)}}{{q\left( A \right)}}{\text{d}}A, \\ = & \underbrace {{{\mathcal{L}_{av}}\left( {p\left( {y{\text{|}}A,m} \right),q\left( A \right)} \right)}}_{{{\text{accuracy}}}} - \underbrace {{KL\left( {q\left( A \right),p(A|m)} \right)}}_{{{\text{complexity}}}}, \\ \end{aligned}$$

where ${\mathcal{L}_{av}}\left( {p\left( {y{\text{|}}A,m} \right),q\left( A \right)} \right)=p(q\left( A \right)|p\left( {y{\text{|}}A,m} \right))$ is referred to as the accuracy (fit of the model to the data) and the second term is the complexity of the model—the distance or KL-divergence between the approximate posterior $q(A|y,m)$ and the priors $p(A|m)$. This divergence may be reduced by adaptation of priors such as in si-PEB, leading to improvement of model quality as assessed by the free energy. The parameters which maximize the free energy are those that provide the most accurate and simplest explanation for the data.

Having estimated a DCM for each subject, we then specified and estimated the group-level PEB model. This uses the same estimation procedure as described above for each subject’s DCMs, except the procedure optimizes the parameters with respect to a group-level free energy (i.e., it finds explanations for the data from all subjects while allowing for parametric random effects at the between-subject level). This provided estimates of the average connection strengths and free energy at the group level.

### Bayesian model reduction (BMR) and model comparison

Having estimated a ‘full’ model, including all free parameters of interest, BMR can be used to rapidly compute the evidence and parameters of ‘reduced’ (alternative) models. Depending on the hypotheses, these reduced models may have different prior constraints on the connectivity parameters (e.g., informed by structural connectivity) or certain connections may be switched off entirely (by fixing them at their prior expectation). Whereas the variational Laplace procedure described above can be computationally expensive (on the scale of minutes to hours), BMR is analytic and computed in seconds using typical computer hardware. This procedure is particularly useful for larger DCMs with more regions and parameters. Crucially, the outcomes of this analytical procedure are stable and generally consistent with conventional DCM estimation (Rosa et al. 2012; Litvak et al. 2015).

Based on Bayes rule, using a generalisation of the Savage–Dickey ratio (Dickey 1971; Rosa et al. 2012), we can derive the posterior distribution of the parameters of the reduced model $p\left( {A{\text{|}}y,{m_R}} \right)$, as well as the relative evidence for the reduced with respect to the full model $~\frac{{p\left( {y{\text{|}}{m_R}} \right)}}{{p\left( {y{\text{|}}{m_F}} \right)}}$:

9 $$\begin{gathered} p\left( {A{\text{|}}y,{m_R}} \right)=p\left( {A{\text{|}}y,{m_F}} \right)\frac{{p\left( {y{\text{|}}{m_F}} \right)p\left( {A{\text{|}}{m_R}} \right)}}{{p\left( {y{\text{|}}{m_R}} \right)p\left( {A{\text{|}}{m_F}} \right)}}, \hfill \\ \frac{{p\left( {y{\text{|}}{m_R}} \right)}}{{p(y|{m_F})}}=\mathop \smallint \nolimits^{} p\left( {A{\text{|}}y,{m_F}} \right)\frac{{p(A|{m_R})}}{{p(A|{m_F})}}{\text{d}}A. \hfill \\ \end{gathered}$$

Under the Laplace approximation, this equation gives rise to simple analytic functions of the posterior parameter means and precisions from the full model and the priors of the reduced model [see Eqs. (9) and (10) of Friston and Penny 2011; Supplementary Methods].

As described above, we specified 405 reduced si-PEB models which only differed in their structurally informed prior covariance matrices, but otherwise shared the same generative model (and thus likelihood) $p(y|A,m)$. BMR afforded comparison of the models with respect to their free energy, as an approximation to their log model evidence (Friston et al. 2007, 2016). Furthermore, we used BMR to compare structurally informed with the full, uninformed DCMs at the individual level.

The log Bayes factor for each model, relative to the worst, was calculated by subtracting its (i.e., the smallest) free energy from each model’s free energy. A difference in free energy of approximately three corresponds to a 95% probability that one model better explains the observed data, and we used this as our threshold for concluding there was ‘strong evidence’ for one model over another (Penny et al. 2004). The log Bayes factors were converted to posterior probabilities in the usual way using a softmax function.

## Results

**Fig. 2:**
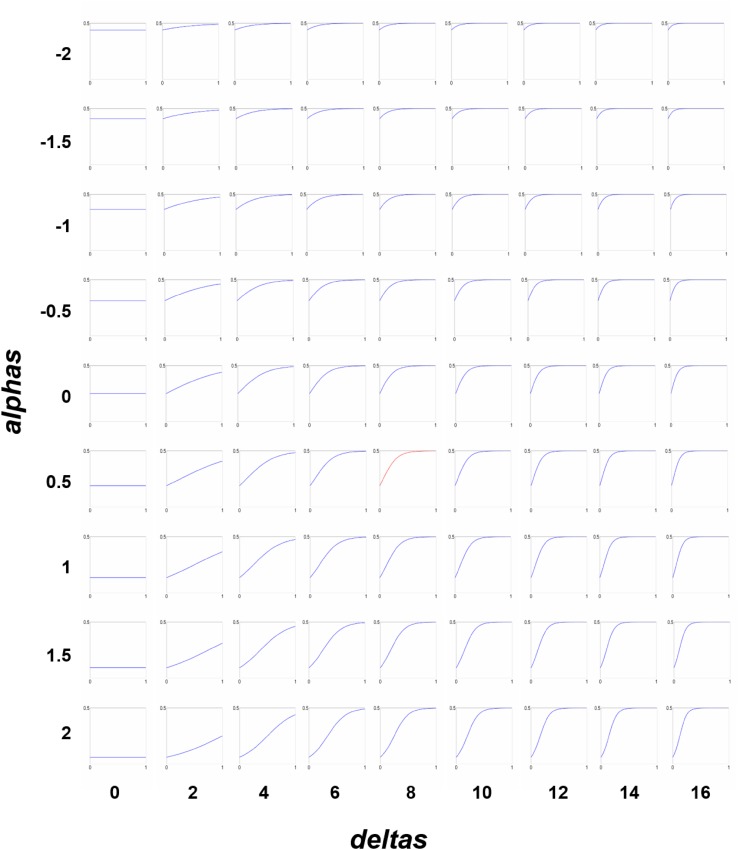
Model space spanned by the hyperparameters *α* and *δ*, shown at ${\Sigma _y}$ = 0.5 for illustrative purposes. The mapping from structural connection strength (*x*-axis in each plot) to prior covariance for effective connectivity (*y*-axis in each plot) is governed by the hyperparameters *α* (range from − 2 to 2) and *δ* (range from 0 to 16). The optimal mapping (*α* = 0.5, *δ* = 8 and ${\Sigma _y}$ = 0.5) yielding the highest posterior probability (see Fig. 3) is highlighted with a red plot

Comparison of 405 group-level models, with different mappings from group structural connection strength to second-level prior variance on effective connectivity, indicated that including structural connectivity significantly improved the model evidence. The highest log model evidence relative to the full, uninformed model equalled 15.52 (considered ‘very strong evidence’; Penny et al. 2004), corresponding to a posterior probability of almost 1.00 that the structural priors improved model evidence. This si-PEB model was obtained with a mapping governed by the hyperparameter combination *α* = 0.5, *δ* = 8, ${\Sigma _{y~\hbox{max} }}$ = 0.5 (Fig. 2). The computation time for PEB estimation and comparing all 405 models using BMR on a PC workstation was 15.5 s. Given around 3460s (58 min) for individual DCM estimation, use of conventional DCM without BMR would have taken approximately 1,401,300 s (389 h) per subject (i.e., 4668 h for the entire group) to invert the 405 different mappings from structural connection strength to effective connectivity priors.

**Fig. 3:**
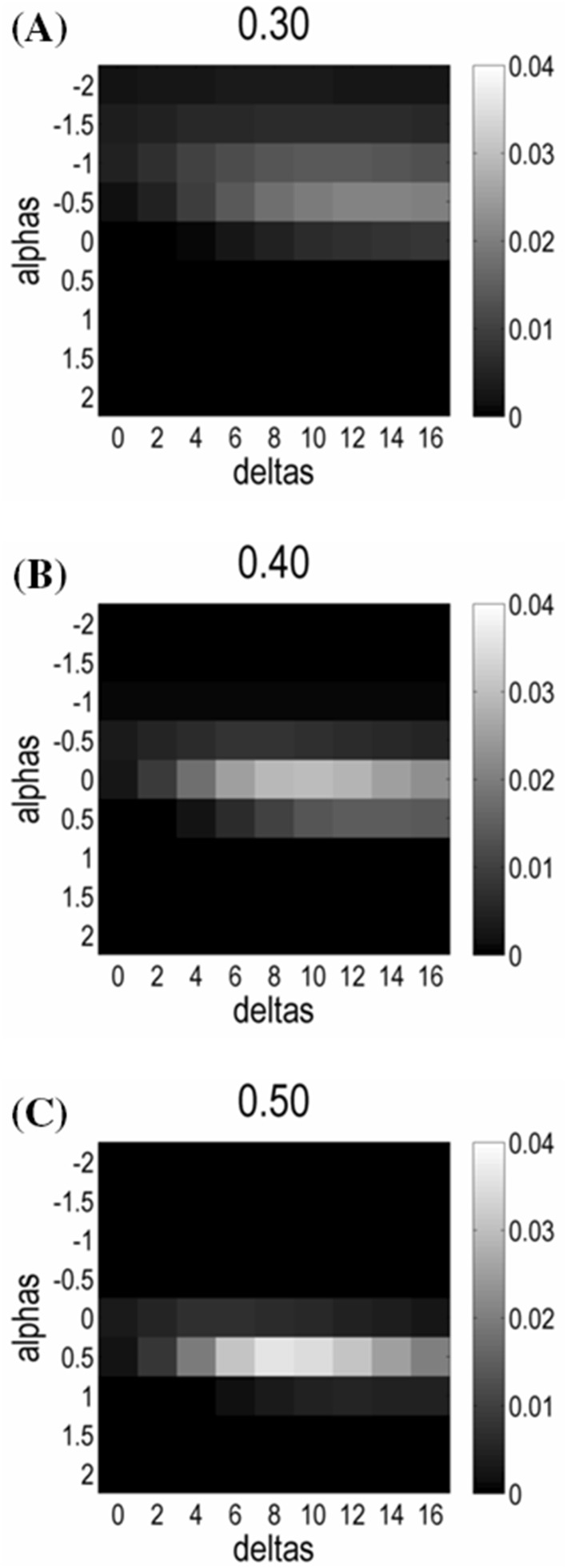
Illustration of posterior probabilities of PEB models with different prior variance of extrinsic effective connections defined by the corresponding structural connection strength, depending on the hyperparameters *α* (*y*-axis) and *δ* (*x*-axis) at the three most probable levels of full prior covariance (**a**${\Sigma _y}$ = 0.3; **b**${\Sigma _y}$ = 0.4; and **c**${\Sigma _y}$ = 0.5). The other two levels (${\Sigma _y}$ = 0.1 and ${\Sigma _y}$ = 0.2) are omitted for illustrative purposes, as the corresponding posterior probabilities are all close to zero. At each level of ${\Sigma _y}$, the optimal range of *α* is from − 0.5 to 0.5, and posterior probability increases from *δ* = 0 (no structural information transmitted to prior PEB covariance) to peak at *δ*-values of 8–10. As can be seen, structurally informed PEB (si-PEB; *δ* > 0) priors outperformed models with structurally uninformed priors (*δ* = 0)

As illustrated in Fig. 3, the log-evidence increased as *δ*-values (i.e., slope of the sigmoid transform from structural connectivity to prior covariance) progressed from 0 (no influence of structural connectivity) to 10. The model probability then diminished towards the upper limit of this hyperparameter range (*δ* = 16). Therefore, PEB with structurally informed priors (*δ* > 0) outperformed models without any influence of structural connectivity (*δ* = 0). The optimal range for α was between − 0.5 and 0.5, and for ${{{\varvec{\Sigma}}}_{y~{\text{max}}}}$ between 0.3 and 0.5.

**Fig. 4:**
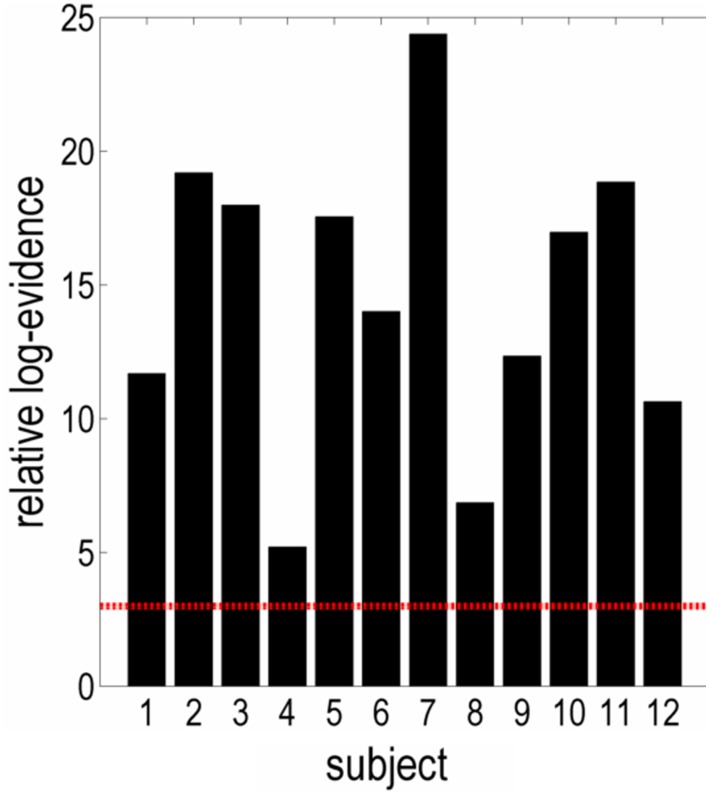
Individual increases in log-evidence for the optimal structurally informed model as compared to the full, uninformed model. These results show strong evidence for structural priors in every subject. Structural constraints were implemented using the optimal group-level mapping (with hyperparameters *α* = 0.5, *δ* = 8 and ${\Sigma _y}$ = 0.5) from structural connectivity to prior variance on individual extrinsic effective connectivity. The relative log-evidence (*y*-axis) represents the difference in evidence between the structurally informed and uninformed (full) baseline model in individual subjects (*x*-axis). The red dashed line indicates a threshold of three that constitutes very strong evidence for one model over another

Inclusion of structural connectivity may not only be useful at the group-level, but also for individual analyses of effective connectivity. To illustrate this, the optimal mapping—from structural connectivity to prior variance on effective connectivity—afforded by the si-PEB approach was applied to single subject DCMs. Bayesian model comparison indicated very strong evidence for the structurally informed models as compared with the uninformed, baseline model in every subject (Fig. 4).

## Discussion

In this work, we introduced an efficient method (si-PEB) for integrating structural and effective connectivity at the group level. This overcomes the high computational cost of identifying the optimal mapping between structure and function in DCM, which was particularly acute for large brain networks. Furthermore, by operating at the group level, high-quality diffusion data can be introduced into effective connectivity models—either from the same subjects or from an atlas, such as the dense structural connectivity matrix due to be released by the Human Connectome Project (Van Essen et al. 2013)—minimizing the potential for local minima caused by noisy individual data. Our procedure, implemented in a simple software function (spm_dcm_peb_si.m), uses BMR to score a large number of group-level models which differ in their structural constraints on effective connectivity. Bayesian model comparison enables selection of the optimal mapping that yields maximal group-level model evidence. We demonstrated this approach for a 12-node network involved in visual body motion processing, finding the optimal structurally informed group-level model of effective connectivity had substantially more evidence than uninformed models. Furthermore, the consistently higher model evidence for structurally informed DCMs in every subject suggests inclusion of structural connectivity in analyses of effective connectivity may also be beneficial at the individual level.

The si-PEB procedure not only confirms, but substantially extends the previous work showing that effective connectivity modelled in DCM is usefully shaped by structural connectivity priors (Stephan et al. 2009). Use of the recent methodological advances BMR and PEB (Friston et al. 2016) allows the si-PEB analysis to run within a few seconds, whereas the previous approach for conventional DCM could take several hours or days (Stephan et al. 2009). Comparative studies demonstrated that BMR provides stable outcomes consistent with conventional DCM (Rosa et al. 2012; Litvak et al. 2015). This makes it tractable for experimenters to include information on structural connectivity as priors in DCM analyses of networks with a large number of regions and connections as a matter of routine. Notably, the use of this approach is not restricted to task-related data but could also be implemented with DCM for resting-state fMRI (Friston et al. 2014; Razi et al. 2015). Importantly, the si-PEB procedure can be used to shape models of effective connectivity based on information other than the direct structural connectivity presented here, such as intra- or extracranial electrophysiological data.

Multimodal integration beyond MRI provides exciting opportunities to consider structural, functional and effective connectivity as distinct yet complementary determinants of functional integration in the brain. For instance, integration of electrophysiology with its exquisite temporal resolution (Bonnefond et al. 2017), data on physical network topography (Pineda-Pardo et al. 2015) as well as analysis and modelling of more fine-grained features such as connectivity within grey matter or mechanisms of synaptic coupling (Breakspear et al. 2003; Leuze et al. 2014; Lo et al. 2015) may open avenues to better conceptualise brain connectivity. Efforts towards generative models of how function evolves from structural connectivity are underway (Ritter et al. 2013; Sanz Leon et al. 2013). DCM also provides a potentially useful framework for developing biophysically plausible forward models for multimodal integration, which would describe how neural circuitry gives rise to both fMRI and electrophysiological data (Friston et al. 2017). Using the si-PEB approach described here, such multimodal models may in future fully capitalise on dMRI, fMRI, and EEG/MEG to jointly inform estimates of neural coupling. Furthermore, intracranial electrophysiology may provide probabilistic atlases describing physiological features of the white-matter pathways subserving effective connectivity (Catenoix et al. 2011; David et al. 2013; Almashaikhi et al. 2014a, b; Donos et al. 2016), which may be introduced as priors in the si-PEB framework developed here. Another interesting extension is assessment of axonal diameter statistics (e.g., *g* ratios: Mohammadi et al. 2015) using microscopic MRI that may provide structural constraints on the axonal conduction delays, which are an important parameter in DCMs of EEG and MEG data.

In terms of the broader application of the si-PEB approach, its utility may vary depending on specific network characteristics. As brain connectivity is underwritten by white-matter pathways, one can expect structural connectivity to shape functional and effective connectivity (Stephan et al. 2009; Pineda-Pardo et al. 2014; Xue et al. 2015; Kang et al. 2017). However, this structure–function relationship is not straightforward and only partially understood. Establishing a probabilistic mapping between structural and effective connectivity acknowledges both this neurobiological perspective and the inherent limitations of the underlying acquisition techniques. Despite sophisticated procedures for imaging and analysis of white matter, and optimisations such as high-field dMRI recordings or diffusion kurtosis imaging (Tournier et al. 2004; Heidemann et al. 2012; Mohammadi et al. 2014), the anatomical accuracy of dMRI remains limited (Thomas et al. 2014; Jbabdi et al. 2015). In particular, fibre crossing or bending challenge the currently available techniques for tract reconstruction based on dMRI (Behrens et al. 2007). This qualification also applies when using HARDI as in the present study. Furthermore, dMRI cannot provide any information on directionality of signalling within fibres or the functional processes the fibres subserve. In turn, as DCM is exclusively based on functional brain data, it cannot be used to distinguish between monosynaptic or polysynaptic connections (Friston et al. 2003). In the absence of detectable direct structural pathways, indirect communication via hidden nodes may mediate apparently direct effective and functional connectivity between brain regions (Sporns et al. 2000; Koch et al. 2002; Friston et al. 2003; Honey et al. 2009; Buckner et al. 2013). As simulated neural activity has been found to correspond to structural connectivity in the macaque cortex at a timescale of minutes but to vary substantially in timeframes of seconds (Honey et al. 2007), there may be further caveats when relating ‘static’ measures of structural connectivity to dynamic effective and functional connectivity. These considerations illustrate why a general, consistent mapping from structural to effective connectivity is unlikely to exist—and motivate a study-specific probabilistic mapping, such as afforded by the approach introduced here. It will then be possible to evaluate how the advantages of structural priors generalise.

Bridging structural and functional brain connectivity could offer particularly valuable insights in clinical neuroscience. Measures of white-matter integrity have been associated to altered functional connectivity in patients with amyotrophic lateral sclerosis (Douaud et al. 2011), high functioning autism (Mueller et al. 2013), schizophrenia (Schlösser et al. 2007; Pomarol-Clotet et al. 2010; Motzkin et al. 2011), bipolar disorder (Motzkin et al. 2011), anorexia and bulimia nervosa (Frank et al. 2016) and temporal lobe epilepsy (Voets et al. 2009). Given the volume of data afforded by multimodal imaging, computational approaches towards integration of structure and function appear indispensable to optimally inform diagnosis and management, as demonstrated by connectivity-based classification of patients with movement disorders (Fratello et al. 2017) and cognitive decline (Pineda-Pardo et al. 2014). Overall, such approaches may afford a more comprehensive understanding of the subtle alterations underlying pathologies of brain structure and function in neurological and psychiatric conditions.

In summary, these considerations speak to integrative approaches to modelling connectivity, which necessarily will draw upon multiple modalities informing on brain structure and function in normalcy and pathology.

## Electronic supplementary material

Below is the link to the electronic supplementary material.

Supplementary material 1 (DOCX 32 KB)
