# Supplementary material for: Linking structural and effective brain connectivity: structurally informed Parametric Empirical Bayes (si-PEB)
Source: Brain Struct Funct. 2018 Oct 9;224(1):205–17. doi: 10.1007/s00429-018-1760-8 (PMC6373362; doi:10.1007/s00429-018-1760-8)
Supplement: Supplementary file 1 — Supplementary material 1 (DOCX 32 KB) [file 429_2018_1760_MOESM1_ESM.docx]

**Supplementary Methods**

**General linear model underlying PEB**

The second level (between-subject) model $\Gamma^{(2)}$ in PEB (Eq. 5) is a GLM with the form:

|  | $\Gamma^{\left( 2 \right)}\left( \theta^{\left( 2 \right)} \right)=\left( X\otimes I_{P} \right)\beta$ | 10. |
| --- | --- | --- |

Where $X\in\mathbb{R}^{S\times C}$ is the design matrix ($S$ is the number of subjects, $C$ is the number of covariates), $I_{P}$ is the identity matrix of dimension $P$ (where $P$ is the number of connection parameters in the DCM) and the operator $\otimes$ is the Kronecker product that duplicates each element of the design matrix for each DCM connectivity parameter. The parameters of the GLM are $\beta\in\mathbb{R}^{CP\times1}\subset\theta^{\left( 2 \right)}$. In this study, we were only interested in the commonalities across subjects (the group mean), so $X$ was a column vector of dimension $S\times1$, with one $\beta$ parameter representing the group mean strength of each connection.

The between-subject variability $\epsilon^{(2)}$ with precision $\Pi^{(2)}$ was parameterized using a single precision parameter $\gamma$:

|  | $\epsilon^{(2)}\sim N(0,\Sigma^{(2)})$  ${\Pi^{\left( 2 \right)}=\Sigma}^{\left( 2 \right)^{-1}}=I_{S}\otimes\left( Q_{0}+e^{-\gamma}Q_{1} \right)$ | 11. |
| --- | --- | --- |

Where $Q_{0}\mathbb{\in R}^{P\times P}$ is the lower bound on precision, which takes on a small positive value, and the precision (hyper)parameter $\gamma\subset\theta^{\left( 2 \right)}$ scales a precision component $Q_{1}\mathbb{\in R}^{P\times P}$. Here, we used the defaults in SPM, which sets $Q_{1}$ to 16 times the prior precision used for any single subject’s connectivity parameters.

**Statistical bases for Bayesian model reduction**

To build the foundation intuition behind BMR, consider Bayes rule for both a full model $m_{F}$ and a reduced model $m_{R}$:

|  | $p\left( \theta\vert y,m_{R} \right)=\frac{p\left( y \vert\theta,m_{R} \right)p\left( \theta\vert m_{R} \right)}{p\left( y \vert m_{R} \right)}$ $p\left( \theta\vert y,m_{F} \right)=\frac{p(y\vert\theta,m_{F})p(\theta\vert m_{F})}{p(y\vert m_{F})}$ | 12. |
| --- | --- | --- |

By rearrangement:

|  | $p\left( \theta\vert y,m_{R} \right)p\left( y \vert m_{R} \right)=p\left( y \vert\theta,m_{R} \right)p\left( \theta\vert m_{R} \right)$  $p\left( \theta\vert y,m_{F} \right)p\left( y \vert m_{F} \right)=p\left( y \vert\theta,m_{F} \right)p\left( \theta\vert m_{F} \right)$ | 13. |
| --- | --- | --- |

If the models differ only in their priors, but are based on the same generative model (and thus same likelihood; [Friston et al. 2016](#_ENREF_1)), then the above equation can be simplified:

|  | $\frac{p\left( \theta\vert y,m_{R} \right)p\left( y \vert m_{R} \right)}{p\left( \theta\vert y,m_{F} \right)p\left( y \vert m_{F} \right)}=\frac{p\left( \theta\vert m_{R} \right)}{p\left( \theta\vert m_{F} \right)}$ | 14. |
| --- | --- | --- |

From Eq. 14, we can derive two quantities of interest (Eq. 9): by re-arrangement we get the posterior distribution over the parameters of the reduced model, and by integrating over the parameters, we get the evidence ratio of the reduced and full models. Analytic expressions for these quantities, using multivariate normal densities (the Laplace approximation) are provided in Friston et al. (2016) and are implemented in the SPM software used here (function **spm_log_evidence_reduce.m**).

Friston KJ, Litvak V, Oswal A, Razi A, Stephan KE, van Wijk BC, Ziegler G, Zeidman P. 2016. Bayesian model reduction and empirical Bayes for group (DCM) studies. Neuroimage. 128:413-431.
